# IMPROVING AWARENESS OF FOOD RESOURCES FOR OLDER ADULTS THROUGH REGIONAL COLLABORATIVE EFFORT

**DOI:** 10.1093/geroni/igae098.3346

**Published:** 2024-12-31

**Authors:** Joan Ilardo, Angela Zell

**Affiliations:** Michigan State University East Lansing, East Lansing, Michigan, United States; Michigan State University East Lansing, East Lansing, Michigan, United States

## Abstract

The Successful Nutrition Programs Across the Lifespan (SNP-AL) created a roadmap with recommendations for selecting sustainable, successful, evidence-informed nutrition programs that meet the community’s needs and foster cross-sector collaboration; implementing interventions; and gathering data to measure program outcomes. The Capital Area Coalition (CAC) addressed lack of communication around its regional food resources. The pandemic changed many community resources, so residents weren’t aware of current food resources or how to access emergency food. 2-1-1 coordinates with regional resources and provides information about local health and human services resources. CAC’s strategy included a media campaign, implemented in Spring and Summer 2023, directing residents with food insecurity to contact 2-1-1 for available resources, using local TV and radio spots and including 1400 leaflets containing information about food resources in produce boxes delivered to income-eligible residents. They provided 2-1-1 information to appropriate agencies. We used the Collaborative Effectiveness Assessment from the Prevention Institute to measure cohesiveness. Results demonstrated CAC was highly motivated with trust, strong communication, and conflict resolution skills. They perceived their mission was simultaneously overarching and comprehensive, aligning with their overall objective. CAC’s sustainability plan for 2-1-1 is helping local agencies recognize the importance of providing current information to 2-1-1. Calls to 2-1-1 was the indicator of impact of their media campaign. We compared data from 2-1-1 for 4/2022-6/2022 and 4/2023-6/2023. Food Lines referrals increased from 32 to 37. Food Stamps/SNAP decreased to 49 from 55. Food Pantries referrals increased the most, increasing 29% from 342 in 2022 to 479 in 2023.

